# Homeostasis of cytoplasmic crowding by cell wall fluidization and ribosomal counterions

**DOI:** 10.21203/rs.3.rs-4138690/v1

**Published:** 2024-04-19

**Authors:** Markus Basan, Avik Mukherjee, Yanqing Huang, Seungeun Oh, Carlos Sanchez, Yu-Fang Chang, Xili Liu, Gary Bradshaw, Nina Benites, Johan Paulsson, Marc Kirschner, Yongjin Sung, Jens Elgeti

**Affiliations:** HARVARD MEDICAL SCHOOL; Harvard Medical School; Harvard Medical School; Harvard Medical School; Harvard Medical School; Harvard Medical School; Harvard Medical School; Harvard Medical School; Harvard Medical School; Harvard University; Harvard Medical School; UW Milwaukee; FZ Juelich

## Abstract

In bacteria, algae, fungi, and plant cells, the wall must expand in concert with cytoplasmic biomass production, otherwise cells would experience toxic molecular crowding^1,2^ or lyse. But how cells achieve expansion of this complex biomaterial in coordination with biosynthesis of macromolecules in the cytoplasm remains unexplained^3^, although recent works have revealed that these processes are indeed coupled^4,5^. Here, we report a striking increase of turgor pressure with growth rate in *E. coli,* suggesting that the speed of cell wall expansion is controlled via turgor. Remarkably, despite this increase in turgor pressure, cellular biomass density remains constant across a wide range of growth rates. By contrast, perturbations of turgor pressure that deviate from this scaling directly alter biomass density. A mathematical model based on cell wall fluidization by cell wall endopeptidases not only explains these apparently confounding observations but makes surprising quantitative predictions that we validated experimentally. The picture that emerges is that turgor pressure is directly controlled via counterions of ribosomal RNA. Elegantly, the coupling between rRNA and turgor pressure simultaneously coordinates cell wall expansion across a wide range of growth rates and exerts homeostatic feedback control on biomass density. This mechanism may regulate cell wall biosynthesis from microbes to plants and has important implications for the mechanism of action of antibiotics^6^.

## Introduction

**Figure 1:**
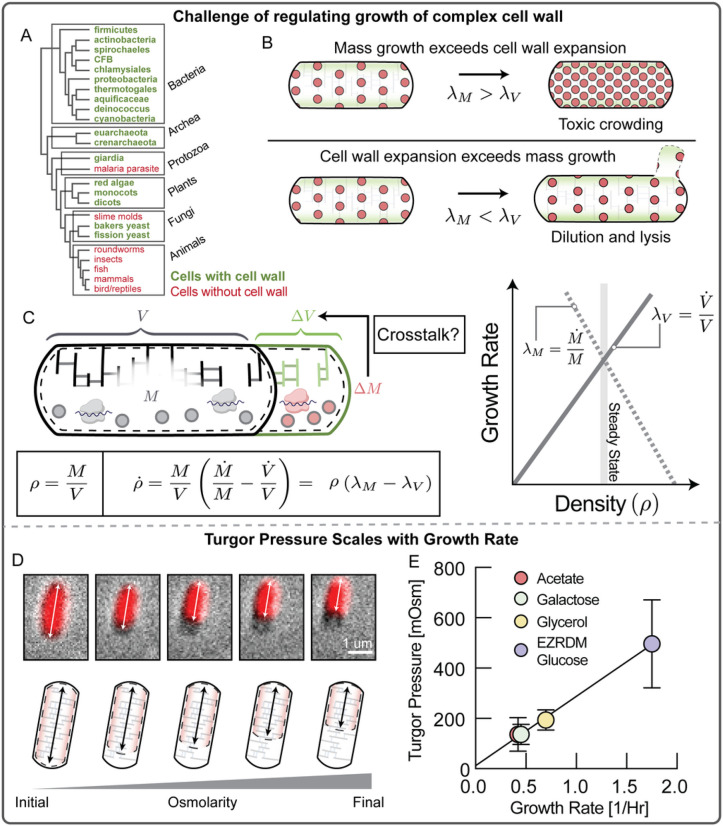
Turgor pressure increases with growth rate. **A,** Cell walls are common across many evolutionary taxa. (drawn after Wikimedia commons) **B,** Cells with cell-wall must coordinate biomass growth with expansion of the cell wall. If biomass growth exceeds volume growth, the result is toxic cytoplasmic crowding. Volume growth with insufficient biomass growth results in cytoplasmic dilution and eventually lysis due to insufficient production of cell wall precursors by cytoplasmic metabolic pathways. **C,** To achieve homeostasis of biomass density, addition of biomass ΔM to the cytoplasm must be coordinated with a corresponding increase in cell volume . The increase in cell volume comes from the expansion of the bacterial cell wall, a chemically complex, load-bearing structure, whereas biomass production occurs in the cytoplasm largely via ribosomal protein synthesis. It is currently unknown how these two fundamental cellular processes are kept in concert and coordinated across a wide range of growth rates. **D,** Osmotic shocks put cells into plasmolysis, a state where the cytoplasm is retracted from the cell wall and osmotic pressure of the cytoplasm is balanced by externally applied osmolarity. Cytoplasmic volume before and after the shift can be measured using a fluorescent cytoplasmic marker. **E,** Turgor pressure in different growth conditions plotted against corresponding growth rates in these conditions. Fits to the Boyle-van't Hoff equation underlying these data, originating from osmotic shocks of different magnitude are presented in Fig. S1. Individual data points in Fig. S1 represent biological replicates and each of these data point results from averaging 13 randomly selected cells undergoing this osmotic shock. Error bars represent uncertainty of the fit of the slope to the data in Fig. S1.

Bacteria are confined by a tough load-bearing cell wall. The cell wall is a complex and resource-intensive biochemical structure that must expand in concert with biomass growth to prevent cell death by lysis or toxic molecular crowding, which inhibits metabolism and other crucial cellular functions (Fig. 1A-C). How cells achieve this coordination remains unknown. A fine-tuned regulatory coordination between cell wall synthesis rates and biomass production rates is thought to exist, but molecular interactions and molecular players that could mediate this coupling have not been discovered^3^.

The bacterial cell envelope is one of the most complex chemical structures in nature and its synthesis and expansion involves myriad biochemical processes. Mis-regulation of either cell wall remodeling or cell wall precursor biosynthesis quickly leads to lysis and death, a vulnerability that is exploited by some of our most important classes of antibiotics. Hence, it is crucial to uncover how two of the most important cellular processes – biomass production in the cytoplasm and biosynthesis and expansion of the cell envelope – are kept in concert and what is the regulatory process of this coordination. Expansion of the cell envelope and biomass production reflect relative rates of surface and volume growth respectively, which have been found to be key determinants of cell size^4^.

Turgor pressure is the osmotic pressure exerted by the cytoplasm on the cell wall; it is reasonable to think that it plays a role in driving cell wall expansion. However, it is unknown how cells regulate turgor pressure and whether turgor pressure is constant or changes with growth rate. It is also unclear if and how turgor pressure is coupled to physiological parameters like cellular biomass. Biomass macromolecules like protein, RNA and DNA are present at relatively low molar concentration inside the cell, meaning that their direct contribution to the osmotic pressure that underlies turgor is insignificant. Surprisingly, experiments in *E. coli* with periodic osmotic shocks that put cells into plasmolysis, a state where turgor pressure vanishes, showed that the cell volume very quickly recovered after cells exited plasmolysis^7^. From this observation, it was concluded that cell wall biosynthesis continues during plasmolysis and therefore that turgor pressure plays no role in cell wall expansion in *E. coli*^7^.

However, this conclusion raises a conundrum: If synthesis of the cell wall were only determined by the flux of cell wall biosynthetic pathways or the abundance specific enzymes in the cell wall, how could cells then regulate the correct expression levels of these proteins, as they encounter dynamic shifts in their environment requiring different growth rates. In natural environments, bacteria experience sudden transitions between hundreds of diverse conditions, many of which are known to require large-scale rearrangement of their entire proteome^8^. Without some stabilizing feedback on cell wall biosynthesis, an exceedingly complicated, fine-tuned regulatory program would be required, and even then, cells would be constantly at risk of lysis due to small fluctuations or mistakes in expression. We therefore postulated that such feedback mechanisms must exist and sought to identify the interactions between biomass growth and cell wall biosynthesis.

## Turgor pressure increases proportional to growth rate

To better understand the physiological role of turgor pressure, we wanted to quantify the growth rate dependence of turgor pressure. Measuring turgor pressure is challenging because pressure exerted by the cytoplasm is directly balanced by the cell envelope^9^ and therefore turgor pressure is not easily accessible. One method to assess the magnitude of turgor pressure involves applying osmotic shocks to bacteria to induce plasmolysis. When bacteria are in plasmolysis, the cytoplasm is compressed and detached from the cell wall at the bacterial poles (Fig. 1D). During plasmolysis, cytoplasmic osmotic pressure is directly balanced by external osmolarity. By measuring the fold-change in cytoplasmic volume as a function of the applied osmolarity under such conditions, it is then possible to calculate turgor pressure of the cell before the osmotic shock (Fig. S1). When we determined turgor pressure of *E. coli* in different growth conditions, we discovered a striking linear increase of turgor pressure with growth rate (Fig. 1E & Fig. S1).

## Scaling of turgor with growth rate is essential for biomass density homeostasis

**Figure 2:**
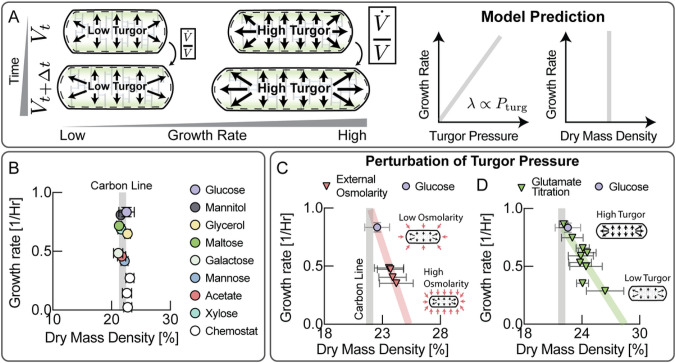
Turgor pressure coordinates cell envelope expansion with biomass growth. **A,** We hypothesized that the physiological role of the observed increase in turgor pressure with growth rate in Fig. 1 is to coordinate growth rate of cell volume with growth rate of biomass. A linear increase of turgor pressure with growth rate ensures that biomass density remains constant across growth rates. **B,** Quantitative phase microscopy measurements show that biomass density is indeed remarkably constant across a wide range of growth conditions. Data for rich defined media is shown in Fig. S5. Growth rates are averages of biological replicates. Error bars in growth rate are the standard deviation from these biological replicates. Plotted dry mass density values result from averaging mean dry mass densities for the measured cell population from different biological replicates. Error bars in dry mass density were determined by propagating the standard deviations of the single cell distributions of the different measurements (Acetate: 3 measurements from n=3 biological replicates; Glucose: 5 measurements from n=4 biological replicates; Glycerol: 6 measurements from n=6 biological replicates; Galactose: 1 measurement from 1 biological replicate; Maltose: 9 measurement from 7 biological replicates; Mannitol: 5 measurements from n=4 biological replicates; Xylose: 3 measurements from n=3 biological replicates; Chemostat conditions: 2 measurements from 2 biological replicates for each data point). Carbon line represents the average dry mass density of different carbon conditions shown in this panel except chemostat experiments **C,** Cells become dense and growth rates slow down when grown in hyperosmotic media that reduce turgor pressure^13^. Individual biological replicates plotted. Error bars are the standard deviation of the single cell distribution of the biological replicate. **D,** Reducing turgor pressure by titration of intracellular glutamate results in higher biomass density and slower growth, similar to the effect of external osmolarity. Individual experiments for different induction levels and biological replicates were binned by their measured growth rate (increments of 0.05/Hr). Error bars were determined by propagating the standard deviation of the single cell distributions from the individual experiments in each bin.

But what is the physiological role of this unexpected coordination of turgor pressure with growth rate? Turgor pressure results in tension in the cell wall and it has been established that mechanical forces can influence cell wall elongation in *E. coli*^10^. Therefore, we surmised that increasing turgor pressure with growth rate could coordinate expansion of the cell envelope with growth rate (Fig. 2A). In this view faster growth requires faster expansion of the cell envelop. It is presently not clear how bacteria achieve this coordination. The most likely explanation which we test is that an increase in turgor pressure observed mediates the rate of cell wall expansion, thereby keeping the biomass density constant at different rates of growth (Fig. 2A).

To test this simple hypothesis, we measured the effect of changing turgor pressure on biomass density. If turgor pressure were indeed essential for driving volume expansion at different growth rates, perturbations of turgor pressure should strongly affect biomass density. We used computationally enhanced QPM (ceQPM)^11^ to simultaneously measure cell mass and volume and (see Fig. S2-4 & Materials and Methods) and found that biomass density is remarkably constant across a large range of growth rates on different food sources (Fig. 2B & Fig. S5), consistent with previous observations ^5,12^. By contrast, hyperosmotic growth conditions that have been shown to reduce turgor pressure^13^, resulted in higher biomass density and slower steady-state growth (Fig. 2C). To control turgor pressure more directly, we genetically titrated glutamate. Glutamate is the most abundant intracellular metabolite with concentrations of about 100mM ^14^. We used a strain developed by the Hwa lab, in which glutamate producing enzymes were placed under an inducible promoter ^8,15^. Indeed, by titrating intracellular glutamate, it was possible to control cellular biomass density (Fig. 2D). The effect was comparable to the effect of higher medium osmolarities (Fig. 2C), consistent with the interpretation that both perturbations affect turgor pressure.

## Cell wall fluidization allows turgor to mediate cell volume expansion

To better understand the control of cell volume growth via turgor pressure, we formulated a simple mathematical model summarized in Box 1 and Supplementary Note 1. Our goal was to find a minimal, coarse-grained description of the rheological material properties of the cell wall, incorporating the effect of mechanics, including the key features of both experimental observations ^4,5^ and previous models^10,16,17^. There is ample experimental evidence that cell wall elongation of *E. coli* can be influenced by mechanical stress ^10,17^. For instance, it has been shown that spatially confining *E. coli* in wells, deformed the bacterial envelope; when the bacteria were removed from the wells, cells maintained this new geometry for some time, before slowly relaxing back to the straight rod shape^10^. Similarly, mechanical forces from fluid flow in microfluidic devices can plastically deform growing *E. coli* cells and make them grow in a curved, rather than straight rod shape ^17^. Mechanical coupling with growth could emerge from cell wall mechano-endopeptidases that remodel the cell wall in a stress-dependent way ^10^. It has been shown theoretically that an elastic material with stress-dependent remodeling behaves like a viscoelastic Maxwell material and can be modeled as a fluid on long timescales ^18^. Moreover, even constant activity of cell wall endopeptidases can result in cell wall fluidization, as illustrated in Box 1 (top), which is similar to previous models of dislocation-mediated growth of the bacterial cell wall ^19^.

We therefore modeled the cell wall as a viscoelastic Maxwell fluid which is elastic on short timescales and as a viscous fluid on long timescales along its axis of elongation^20^. As illustrated in Box 1 (bottom), this viscoelastic model naturally results in a volume expansion rate $\dot{V}∕V$ that is directly proportional to turgor pressure. Thus, if turgor pressure is proportional to growth rate, as we observe experimentally (Fig. 1), this is precisely the dependence required for a pacemaker of cell volume growth to render dry mass density constant across different growth rates (see Fig. 2A & Supplementary Note 1, Eq. [S5]).

## Cell wall fluidization affects cell width, biomass density and growth rate

**Figure 3:**
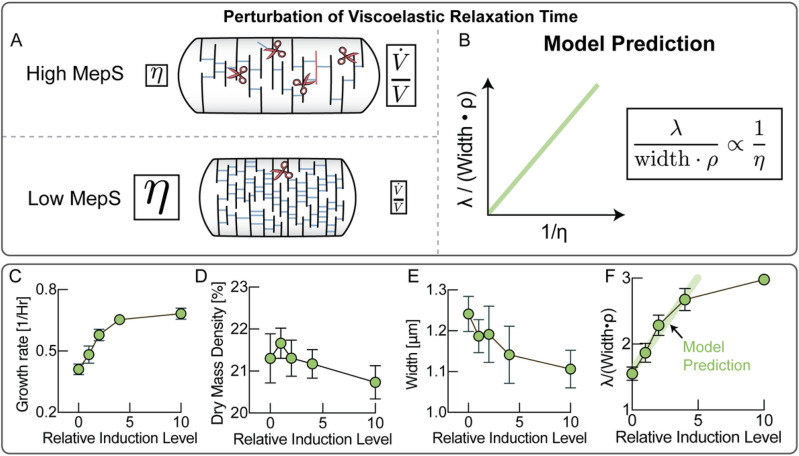
Cell wall viscosity can be controlled by endopeptidase titration. **A,** Titration of the abundance of cell wall endopeptidases affects the effective viscosity of the cell wall, resulting in a slower volume growth rate with lower expression levels. **B,** the model predicts that increasing cell wall viscosity must be compensated by a combination of growth rate, biomass density and cell width (see Supplementary Note 1, Eq. [S13]). **C,** Growth rate as a function of induction levels of endopeptidase expression (number of biological replicates per induction level: 0: 6 replicates; 1: 6 replicates; 2: 5 replicates; 4: 4 replicates; 10: 6 replicates. Error bars represent standard deviation). **D,** Biomass density as a function of induction levels of endopeptidase expression. Biological replicates identical to panel C. Data points are mean of replicates and error bars standard deviations. **E,** Cell width as a function of induction levels of endopeptidase expression. Biological replicates identical to panel C. Data points are mean of replicates and error bars standard deviations. **F,** Comparison of experimental data to model prediction. A non-zero y-intercept can emerge from the activity of other cell wall endopeptidases. Error bars are standard deviations of the values calculated from the mean of individual biological replicates. Biological replicates identical to panel C.

Beyond simply recapitulating our experimental findings, this model makes a set of non-trivial predictions of how cell wall properties and cell shape affect biomass density. These we tested experimentally. According to this model, cell wall rheology on long times is determined by an effective viscosity, given by η=Eτ, where E is the elastic modulus of the elastic network of the cell wall and τ is the viscoelastic relaxation time, which reflects cell wall remodeling by endopeptidases. Therefore, one way to affect cell wall viscosity is changing the rate of remodeling of the cell wall due to endopeptidase limitation ^18^. As illustrated in Fig. 3A, downregulating the abundance of cell wall endopeptidases should result in higher cell wall viscosity, slowing down volume growth rate, according to Box1, Eq. [3]. Higher viscosity from lower hydrolase abundance must thus be compensated by a combination of slower growth rates, greater cell widths or higher dry mass densities, as reflected in the model prediction (Fig. 3B & Supplementary Note 1, Eq. [S13]).

Experimentally, controlling endopeptidase activity is challenging because there are many redundant cell wall hydrolases in *E. coli.* Fortunately, recent work identified three cell wall hydrolases that together are essential for cell growth of *E coli*^21^. We knocked out two of these hydrolases (MepM, MepH) and replaced the chromosomal promoter of the third (MepS) by a linearly inducible expression system ^22^. Indeed, at low induction levels, growth rate depended strongly on MepS induction levels (Fig. 3C). As expected from the model, low hydrolase expression also resulted in denser cells (Fig. 3D) with larger cell widths (Fig. 3E). Plotted in combination, these data are in agreement with the model prediction over the linear induction range (Fig. 3F).

## Cell wall elasticity affects cell wall expansion rate

**Figure 4:**
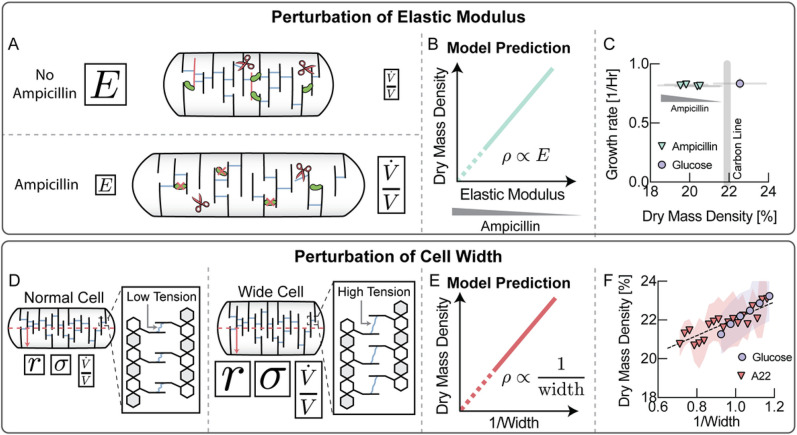
Cell wall elasticity and cell width alter biomass density. **A,** Ampicillin affects the crosslinking density of the peptidoglycan network and thereby its elastic modulus. The elastic modulus of the cell wall is proportional to its effective viscosity and therefore controls volume growth rate. **B,** The model predicts a direct proportionality between biomass density and the elastic modulus of the cell (see Supplementary Note 1, Eq. [S10]). **C,** Sublethal doses of ampicillin resulted in decreasing biomass densities, while growth rate was unaffected. Error bars represent standard deviation of DMD from the distribution cells measured in each experiment. **D,** A larger cell width results in higher cell wall tension and therefore a higher volume expansion rate. **E,** The model predicts that faster volume expansion rate due to higher cell width must be compensated by a drop in biomass density (see Supplementary Note 1, Eq. [S8]). **F,** Average biomass density of individual cells binned by their inverse cell width drops with increasing width. The effect is amplified by inhibiting MreB using A22 and also holds when pooling data across different carbon sources (see Fig. S7). Glucose bin increment: 0.05μm^−1^. A22 bin increment: 0.025μm^−1^. Shaded areas represent the standard deviation of the distribution of cells in each bin.

The effective viscosity of a Maxwell material is not only determined by the rate of remodeling (Fig. 3), but also by the elastic modulus of the underlying elastic network. Therefore, according to the model, affecting the elastic modulus of the cell wall should directly affect cell wall expansion rate and biomass density. Ampicillin is a beta-lactam antibiotic that blocks cell wall crosslinking and insertion of new material into the cell wall ^23^. Ampicillin treated cells have a decreased crosslinking density and a softer, more elastic cell wall (smaller elastic modulus E and effective viscosity η, see Fig. 4A). According to Box1, Eq. [3], this decrease in effective viscosity would be expected to cause a faster volume expansion rate (Fig. 4B & Supplementary Note 1, Eq. [S10]). Indeed, we found that increasing sub-lethal concentrations of ampicillin continuously decreased cellular dry mass density during steady-state growth (Fig. 4C), while growth rates were approximately constant.

## Inverse relationship between cell width and biomass density

Biomass density is thought to be constant and tightly controlled. The model also makes a surprising prediction regarding the effect of cell width on biomass density (Fig. 4D). According to Box1, Eq. [3], volume growth rate of the cell should be directly affected by cell width because turgor pressure creates tension in the cell wall by acting on the cellular cross section. This force scales quadratically with the width, which is distributed along the circumference and scales linearly with cell width, generating tension in the cell wall. Therefore, cell wall tension and the expansion rate of the cell should increase linearly with cell width. Because growth rate is limited by biomass production, higher tension from increasing widths must be compensated by lower turgor pressure. Assuming that turgor pressure is proportional to biomass density (an assumption that will be justified below), the model predicts a simple inverse proportionality between biomass density and width (Fig. 4E & Supplementary Note 1, Eq. [S8]). Indeed, in qualitative agreement with the model, binning cells in a growing population on glucose minimal medium according to their width, we confirmed this surprising dependence of biomass density on cell width (Fig. 4F, blue circles). This single-cell trend was also seen by averaging hundreds of thousands of cells in natural growth conditions based on cell width (Fig. S7). When we inhibited MreB, a protein known to be involved in regulation of cell width in *E. coli*^24,25^, we found a similar, but amplified relationship between biomass density and cell width (Fig. 4F, red triangles). These data demonstrate that cell width indeed exerts a surprisingly strong effect on cellular biomass density, as predicted by the model (Box 1).

## Turgor pressure is generated and regulated by ribosomal counterions

**Figure 5:**
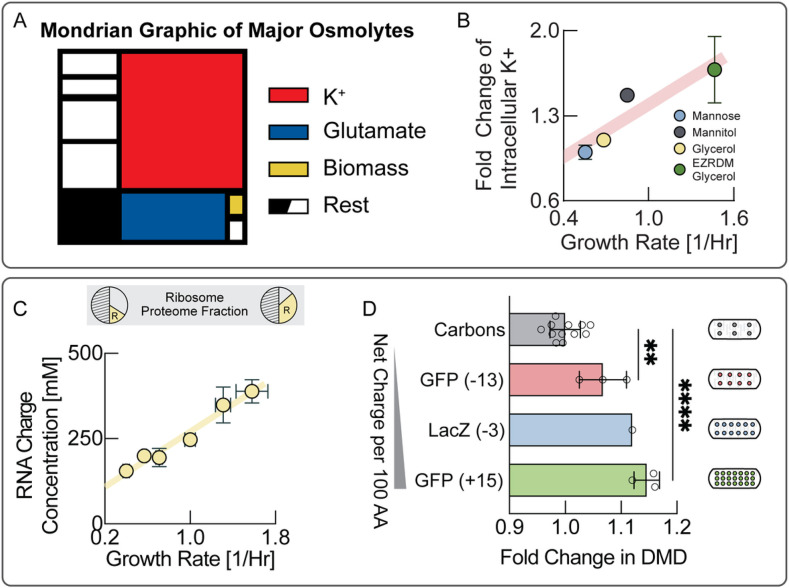
Ribosomal counterions generate turgor to control cell wall expansion. **A,** Schematic illustration of major cellular osmolytes. Potassium and glutamate contribute most to osmotic pressure, whereas the direct contribution from biomass is relatively minor. **B,** Intracellular potassium measured using inductively coupled plasma mass spectrometry (ICP-MS) in low potassium medium in different growth condition. Each data point is determined from several measurements along the growth curve (see Fig. S8 for individual measurements, two biological replicates for each condition). Note that potassium diffuses out of the cell during the mandatory washing step with potassium free medium. This means that we cannot infer absolute intracellular concentrations and only relative changes of intracellular potassium across conditions. Error bars represent uncertainty of the fit in Fig. S8. **C,** RNA/protein ratio changes with growth rate according to growth laws. We converted RNA/protein measurements, including error bars, from Scott et al.^26^ to charge concentrations using total protein and total dry mass measurements from Basan et al.^12^ (see Fig. S9), as well as dry mass density measurements from this work. Counterions balancing net charge of RNA are sufficient to account for the change in turgor pressure across growth rates. **D,** Biomass density increases continuously with overexpression of more positively charged proteins demonstrating the central role of charge balance for biomass density homeostasis. RNA carries one negative charge per nucleotide, whereas proteins are much less negatively charged. Therefore, there is an increase in biomass density in all conditions, but its magnitude depends on net protein charge. Because protein overexpression results in very long cells of irregular shape, biomass densities were determined using Threshold Iterative Volume (TIV) method (see Materials and Methods). Biological replicates shown as individual data points. Error bars are standard deviations from these biological replicates. P-values: **: 0.0042; ****: <0.0001.

So far, our results indicate that an increase in turgor pressure with growth rate (Fig. 1) is indeed crucial for homeostasis of biomass density across growth rates (Fig. 2), enabled by endopeptidase-mediated cell wall fluidization (Fig. 3). But how is this increase in turgor pressure controlled and regulated? Turgor pressure is an osmotic pressure, directly determined by molarities of osmolytes (Fig. 5A). Potassium is by far the most abundant cellular osmolyte in *E. coli* with estimated concentrations surpassing the combined concentrations of all other intracellular osmolytes ^13^ and presumably is the biggest contributor to turgor pressure. To test if the observed increase in turgor pressure originates from changes in potassium concentrations, we measured intracellular potassium on different substrates using mass spectrometry. Indeed, we found a striking linear increase in intracellular potassium with growth rate (Fig. 5B).

These data suggest that the observed increase in turgor pressure (Fig. 1) is likely due to an increase in intracellular potassium. But how does the cell regulate intracellular potassium in precise coordination with growth rate? Potassium is positively charged, while the net charge of the cytoplasm must be neutral ^27,28^. Therefore, growth-rate dependent potassium concentrations must be balanced by an equivalent negative growth-rate dependent charge concentration in the cytoplasm. The concentration of the major intracellular anion glutamate has been shown to be roughly constant across growth rates ^29^ and thus cannot account for potassium charge balance across growth rates. However, an even bigger contributor to cytoplasmic charge comes from the potassium ions necessary to balance cellular RNA, as each nucleotide carries a negative elementary charge. Moreover, cellular RNA content as a fraction of biomass composition is well-known to increase with increasing growth rates, as measured by RNA to protein ratio ^26^. This is largely the result of an increase in ribosomal RNA. When we converted the RNA to protein ratios across growth rates, measured by Scott et al. ^26^, to an intracellular charge concentration by using our own measurements of biomass density, combined with earlier measurements of total protein and total dry mass across growth conditions ^12^ (see Fig. S9), we indeed found that the resulting charge concentrations presented in Fig. 5C, were sufficient to account for the observed growth-rate dependent increase in turgor (Fig. 1C). We also estimated the net charge of ribosomal proteins and confirmed that their net charge constitutes a tiny fraction of the net charge from ribosomal RNA (Table S1).

Based on these data, we propose that an increase in turgor pressure in responses to an increase in growth rate is mediated by the increase in ribosomal RNA and the corresponding retention of potassium in the cytoplasm as RNA counterions, dictated by the requirement for charge balance. We previously proposed that biomass counterions may contribute to dry mass density homeostasis ^12^ and we realized that the contribution from ribosomal RNA, shown in Fig. 5C, far outweighs the contribution from other biomass components in *E. coli.* To test this hypothesis experimentally, we asked how a change in cytoplasmic net charge is reflected in cellular biomass density. If charge balance were indeed the key determinant of turgor driving envelope expansion, then overexpressing large quantities of proteins of different net charge should affect charge balance and be directly reflected in changes in cellular biomass density. More positively charged proteins should result in a larger increase in biomass density. To test this prediction, we used an established system for expression of large quantities of useless proteins ^30^ to overexpress proteins of different net charge, including positively and negatively supercharged versions of GFP, developed by the Liu lab ^31^. Indeed, we find that biomass density continuously increases with the net positive charge per amino acid of the overexpressed protein (Fig. 5D & Fig. S10), as expected if turgor were controlled by charge balance.

## Electro-osmotic model of turgor pressure

The picture that emerges from these data is that turgor pressure is generated and regulated via counterions of negatively charged biomass, with the largest contribution coming from ribosomal RNA, as illustrated in Box 2A. Hence, cell controls turgor pressure by controlling the cytoplasmic concentration of ribosomes. No fine-tuned regulation of ion transport is required in this picture. Applying a previously formulated electro-osmotic model of the cytoplasm^32^, we wanted to test if bacteria could achieve scaling of turgor pressure proportional to ribosomes, across a range of different osmolarities of the medium (Box 2B). Indeed, assuming constant active import of potassium and active export of all other inorganic ions (Box 2B), based on observed intracellular concentrations in *E. coli*^13^, we found a large parameter regime where substantial turgor pressure is generated by ribosomal RNA, which is modulated with changing growth rates via the proteome fraction of ribosomal RNA (Box 2C). The model also shows that bacteria can generate sufficient turgor pressure to drive growth over a large range of media osmolarities (Box 2C, different curves). Changes in medium osmolarity result in changes in turgor and can be compensated by a combination of changes in cytoplasmic biomass density and changing growth rates, consistent with experimental observations for growth at higher medium osmolarities (Fig. 2C). Together, these data suggest that rather than being directly sensed an controlled, turgor pressure is an emergent property that is modulated by environmental factors like medium composition.

## Biomass density homeostasis and regulation of cell wall biosynthesis via ribosome-controlled turgor pressure

By controlling turgor pressure via ribosomal RNA, bacteria elegantly achieve homeostatic feedback control of biomass density and at the same time coordinate cell wall expansion with growth rate: Because turgor pressure is proportional to biomass density (Box 2, Eq. (4)), denser cells have higher turgor pressure due to a higher counterion concentration. This results in faster volume expansion (Box1, Eq. (3)), together constituting a negative homeostatic feedback loop, controlling biomass density (Box 2D).

On the other hand, growth laws require a higher ribosome content with increasing growth rates to support efficient self-replication. Because RNA is a major component of ribosomes, faster growing cells contain substantially more RNA as a fraction of their biomass^26^. This increase of ribosomal RNA automatically results in an increase in turgor pressure due to a larger fraction of ribosomal RNA per biomass (Box 2E & Eq. (4)). Higher turgor then results in faster cell wall expansion (Box1, Eq. (3)), which is precisely the requirement for maintaining constant biomass density across growth rates (Fig. 2 & Eq. (S5)). Thus, the cell achieves both homeostatic feedback control of biomass density, as well as constancy of biomass density across a large range of growth rates, without the need for fine-tuned regulatory coordination between cell wall synthesis and biomass production rates.

## Response to short-term periodic osmotic shocks

The coupling between biomass and turgor pressure given by Box 2, Eq. (4) explains the observation that after short osmotic shocks that put cells into plasmolysis, cell length quickly recovered^7^. These findings can be explained from the observation that biomass growth proceeds during plasmolysis. The feedback loop given in Fig. 6, restores biomass density to its previous level and therefore cell volume quickly recovers. Hence, rather than concluding that turgor is not required for cell wall expansion^7^, the fast recovery of cell volume occurs because turgor pressure drives cell wall expansion and is generated by cellular biomass.

This relaxation can occur quickly, and the detailed temporal dynamics of this relaxation depend on cell wall rheology. In a viscoelastic Maxwell rheology (Eq. (1)), a very fast initial elastic relaxation is followed by a slower viscous relaxation phase. More experimental work with high temporal resolution and many cells, including systematic osmotic shifts of different magnitudes and durations are needed to detect these subtle differences in relaxation dynamics and calibrate parameters of cell wall rheology.

## Discussion

Biomass production and cell wall synthesis are two seemingly disjoint cellular processes that must nevertheless be tightly physiologically coordinated to ensure efficient growth and most likely, survival. Without such coordination cells would produce either toxic conditions due to molecular overcrowding, or lyse from a buildup of turgor pressure or thinning of the cell wall network. It is also important to note that a substantial fraction of cellular resources is devoted to synthesis of the bacterial cell wall^33^. Thus, for optimal growth, synthesis of cell wall precursors must be coordinated with biomass synthesis. It has long been unclear how bacteria achieve this remarkable balance. Our work shows that cell wall expansion is in fact intimately coupled with biomass synthesis, as turgor pressure generated by increased ribosome concentrations sets the pace of volume growth. Metabolic pathways of cell wall biosynthesis can then be regulated by simple product inhibition from cell wall building blocks accumulating in the cytoplasm if they cannot be inserted in the cell wall, thus matching the biosynthetic flux of cell wall building blocks to the requirements from cell wall expansion.

Interestingly, the role of turgor pressure in coordinating cell wall expansion is consistent with our recent findings that starving bacteria expend the lion’s share of their ATP maintenance budget to maintain plasmolysis by exporting ions to reduce turgor ^34^. Why would cells need to maintain plasmolysis for their survival? Plasmolysis is a state with vanishing turgor pressure and according to the pacemaker model of cell volume expansion (Box1, Eq. (3)), vanishing turgor is required to stop the expansion of the cell envelope. Hence, even a small buildup of turgor due to the intracellular concentration of macromolecules, metabolites or the Gibbs-Donnan effect^35^ would result in uncontrolled cell volume expansion in starving bacteria. In starvation conditions, there is no cell wall precursor synthesis, required to reinforce the expanding cell wall. Therefore, expansion would quickly result in lysis and cell death. Indeed, this is precisely the phenotype that we observed in starving bacteria after loss of ion homeostasis due to lack of ATP prior to cell death ^34^.

In conclusion, these simple, yet elegant mechanisms of coordination play a central role in cell physiology. While we focused on the model organism *E. coli* in this work, these mechanisms are likely conserved across evolution, extending to other microbial species. Even cancer cells are frequently addicted to ribosome biogenesis^36^ and can exert substantial forces on their microenvironment^37^. These forces may originate in ribosome-generated cytoplasmic pressure, enabling aggressive expansion in a competition for space inside healthy tissues^38-41^.

## References

[1] Klumpp S., Scott M., Pedersen S. & Hwa T. Molecular crowding limits translation and cell growth. Proceedings of the National Academy of Sciences 110, 16754–16759 (2013). 10.1073/pnas.1310377110 PMC3801028 24082144

[2] Lee A. J. Robust, linear correlations between growth rates and β-lactam–mediated lysis rates. Proceedings of the National Academy of Sciences 115, 4069–4074 (2018). 10.1073/pnas.1719504115 PMC5910845 29610312

[3] van den Berg J., Boersma A. J. & Poolman B. Microorganisms maintain crowding homeostasis. Nat Rev Microbiol 15, 309–318 (2017). 28344349 10.1038/nrmicro.2017.17

[4] Harris L. K. & Theriot J. A. Relative rates of surface and volume synthesis set bacterial cell size. Cell 165, 1479–1492 (2016). 27259152 10.1016/j.cell.2016.05.045 PMC4933963

[5] Oldewurtel E. R., Kitahara Y. & Teeffelen S. van. Robust surface-to-mass coupling and turgor-dependent cell width determine bacterial dry-mass density. Proceedings of the National Academy of Sciences 118, (2021). 10.1073/pnas.2021416118 PMC8364103 34341116

[6] Demain A. L. & Blander R. P. The beta-lactam antibiotics: past, present, and future. Antonie Van Leeuwenhoek 75, 5–19 (1999). 10422578 10.1023/a:1001738823146

[7] Rojas E., Theriot J. A. & Huang K. C. Response of Escherichia coli growth rate to osmotic shock. Proc Natl Acad Sci U S A 111, 7807–7812 (2014). 24821776 10.1073/pnas.1402591111 PMC4040581

[8] Hui S. Quantitative proteomic analysis reveals a simple strategy of global resource allocation in bacteria. Mol Syst Biol 11, (2015). 10.15252/msb.20145697 PMC4358657 25678603

[9] Rojas E. R. & Huang K. C. Regulation of microbial growth by turgor pressure. Curr Opin Microbiol 42, 62–70 (2018). 29125939 10.1016/j.mib.2017.10.015

[10] Wong F. Mechanical strain sensing implicated in cell shape recovery in Escherichia coli. Nat Microbiol 2, 17115 (2017). 28737752 10.1038/nmicrobiol.2017.115 PMC5540194

[11] Liu X., Oh S., Peshkin L. & Kirschner M. W. Computationally enhanced quantitative phase microscopy reveals autonomous oscillations in mammalian cell growth. Proceedings of the National Academy of Sciences 117, 27388–27399 (2020). 10.1073/pnas.2002152117 PMC7959529 33087574

[12] Basan M. Inflating bacterial cells by increased protein synthesis. Mol Syst Biol 11, 836 (2015). 26519362 10.15252/msb.20156178 PMC4631207

[13] Cayley D. S., Guttman H. J. & Record M. T. Biophysical Characterization of Changes in Amounts and Activity of Escherichia coli Cell and Compartment Water and Turgor Pressure in Response to Osmotic Stress. (2000). 10.1016/s0006-3495(00)76726-9 PMC1300771 10733957

[14] Gerosa L. Pseudo-transition Analysis Identifies the Key Regulators of Dynamic Metabolic Adaptations from Steady-State Data. Cell Syst 1, 270–282 (2015). 27136056 10.1016/j.cels.2015.09.008

[15] You C. Coordination of bacterial proteome with metabolism by cyclic AMP signalling. Nature 500, 301–6 (2013). 23925119 10.1038/nature12446 PMC4038431

[16] Huang K. C., Mukhopadhyay R., Wen B., Gitai Z. & Wingreen N. S. Cell shape and cell-wall organization in Gram-negative bacteria. Proc Natl Acad Sci U S A 105, 19282–19287 (2008). 19050072 10.1073/pnas.0805309105 PMC2592989

[17] Amir A., Babaeipour F., McIntosh D., Nelson D. & Jun S. Bending forces plastically deform growing bacterial cell walls. Proc Natl Acad Sci U S A 111, (2014). 10.1073/pnas.1317497111 PMC4000856 24711421

[18] Ranft J. Fluidization of tissues by cell division and apoptosis. Proc Natl Acad Sci U S A 107, 20863–20868 (2010). 21078958 10.1073/pnas.1011086107 PMC3000289

[19] Amir A. & Nelson D. R. Dislocation-mediated growth of bacterial cell walls. Proc Natl Acad Sci U S A 109, 9833–9838 (2012). 22660931 10.1073/pnas.1207105109 PMC3382501

[20] Kruse K., Joanny J. F., Jülicher F., Prost J. & Sekimoto K. Generic theory of active polar gels: a paradigm for cytoskeletal dynamics. The European Physical Journal E 16, 5–16 (2005). 10.1140/epje/e2005-00002-5 15688136

[21] Singh S. K., SaiSree L., Amrutha R. N. & Reddy M. Three redundant murein endopeptidases catalyse an essential cleavage step in peptidoglycan synthesis of *Escherichia coli* K12. Mol Microbiol 86, 1036–1051 (2012). 23062283 10.1111/mmi.12058

[22] Klumpp S., Zhang Z. & Hwa T. Growth rate-dependent global effects on gene expression in bacteria. Cell 139, 1366–75 (2009). 20064380 10.1016/j.cell.2009.12.001 PMC2818994

[23] Kapoor G., Saigal S. & Elongavan A. Action and resistance mechanisms of antibiotics: A guide for clinicians. Journal of Anaesthesiology Clinical Pharmacology vol. 33 300–305 Preprint at 10.4103/joacp.JOACP_349_15 (2017). 29109626 PMC5672523

[24] Hussain S. MreB filaments align along greatest principal membrane curvature to orient cell wall synthesis. Elife 7, (2018). 10.7554/eLife.32471 PMC5854468 29469806

[25] Van Teeffelen S. The bacterial actin MreB rotates, and rotation depends on cell-wall assembly. Proc Natl Acad Sci U S A 108, 15822–15827 (2011). 21903929 10.1073/pnas.1108999108 PMC3179079

[26] Scott M., Gunderson C. W., Mateescu E. M., Zhang Z. & Hwa T. Interdependence of cell growth and gene expression: origins and consequences. Science 330, 1099–102 (2010). 21097934 10.1126/science.1192588

[27] Hille B. Ion channels of excitable membranes. in (2001).

[28] Ikeda T. P., Shauger A. E. & Kustu S. Salmonella typhimuriumApparently Perceives External Nitrogen Limitation as Internal Glutamine Limitation. J Mol Biol 259, 589–607 (1996). 8683567 10.1006/jmbi.1996.0342

[29] Kochanowski K. Global coordination of metabolic pathways in Escherichia coli by active and passive regulation. Mol Syst Biol 17, e10064 (2021). 33852189 10.15252/msb.202010064 PMC8045939

[30] Basan M. Overflow metabolism in Escherichia coli results from efficient proteome allocation. Nature 528, (2015). 10.1038/nature15765 PMC4843128 26632588

[31] Thompson D. B., Cronican J. J. & Liu D. R. Engineering and identifying supercharged proteins for macromolecule delivery into mammalian cells. Methods Enzymol 503, 293–319 (2012). 22230574 10.1016/B978-0-12-396962-0.00012-4 PMC3505079

[32] Mukherjee A. Membrane potential mediates an ancient mechano-transduction mechanism for multi-cellular homeostasis. *bioRxiv* 2023.11.02.565386 (2023) doi:10.1101/2023.11.02.565386.

[33] Neidhardt F. C., 1931-, Ingraham J. L. & Schaechter Moselio. Physiology of the bacterial cell. (1990).

[34] Schink S. The energy requirements of ion homeostasis determine the lifespan of starving bacteria. *bioRxiv* 2021.11.22.469587 (2022) doi:10.1101/2021.11.22.469587.

[35] Donnan F. G. Theorie der Membrangleichgewichte und Membranpotentiale bei Vorhandensein von nicht dialysierenden Elektrolyten. Ein Beitrag zur physikalisch-chemischen Physiologie. Zeitschrift für Elektrochemie und angewandte physikalische Chemie 17, 572–581 (1911).

[36] Catez F. Ribosome biogenesis: An emerging druggable pathway for cancer therapeutics. Biochem Pharmacol 159, 74–81 (2019). 30468711 10.1016/j.bcp.2018.11.014

[37] Helmlinger G., Netti P. A., Lichtenbeld H. C., Melder R. J. & Jain R. K. Solid stress inhibits the growth of multicellular tumor spheroids. Nature Biotechnology 1997 15:8 15, 778–783 (1997). 10.1038/nbt0897-778 9255794

[38] Basan M., Risler T., Joanny J.-F., Sastre-Garau X. & Prost J. Homeostatic competition drives tumor growth and metastasis nucleation. HFSP J 3, 265–272 (2009). 20119483 10.2976/1.3086732 PMC2799988

[39] Levayer R. Solid stress, competition for space and cancer: The opposing roles of mechanical cell competition in tumour initiation and growth. Seminars in Cancer Biology Preprint at 10.1016/j.semcancer.2019.05.004 (2019). PMC7221353 31077845

[40] Moreno E., Valon L., Levillayer F. & Levayer R. Competition for Space Induces Cell Elimination through Compaction-Driven ERK Downregulation. Current Biology 29, 23–34.e8 (2019). 30554899 10.1016/j.cub.2018.11.007 PMC6331351

[41] Levayer R., Dupont C. & Moreno E. Tissue Crowding Induces Caspase-Dependent Competition for Space. Curr Biol 26, 670–7 (2016). 26898471 10.1016/j.cub.2015.12.072 PMC4791483

